# Supplementary material for: Free ferrous ions sustain activity of mammalian stearoyl-CoA desaturase-1
Source: J Biol Chem. 2023 Jun 7;299(7):104897. doi: 10.1016/j.jbc.2023.104897 (PMC10359943; doi:10.1016/j.jbc.2023.104897)
Supplement: Supporting Figures S1–S6 [file mmc1.docx]

**Supporting Information**

**Free ferrous ions sustain activity of mammalian stearoyl-CoA desaturase-1**

Jiemin Shen^1^, Gang Wu^2,*^, Brad S. Pierce^3^, Ah-Lim Tsai^2,*^, Ming Zhou^1,*^

^1^Verna and Marrs McLean Department of Biochemistry and Molecular Biology, Baylor College of Medicine, Houston, TX 77030, USA.

^2^Department of Internal Medicine, University of Texas McGovern Medical School, Houston, TX 77030, USA.

^3^Department of Chemistry & Biochemistry, University of Alabama, Tuscaloosa, AL 35487, USA.

^*^Correspondence to Ming Zhou ([mzhou@bcm.edu](mailto:mzhou@bcm.edu)), Gang Wu ([gang.wu@uth.tmc.edu](mailto:gang.wu@uth.tmc.edu)), and Ah-Lim Tsai ([Ah-Lim.Tsai@uth.tmc.edu](mailto:Ah-Lim.Tsai@uth.tmc.edu))

**
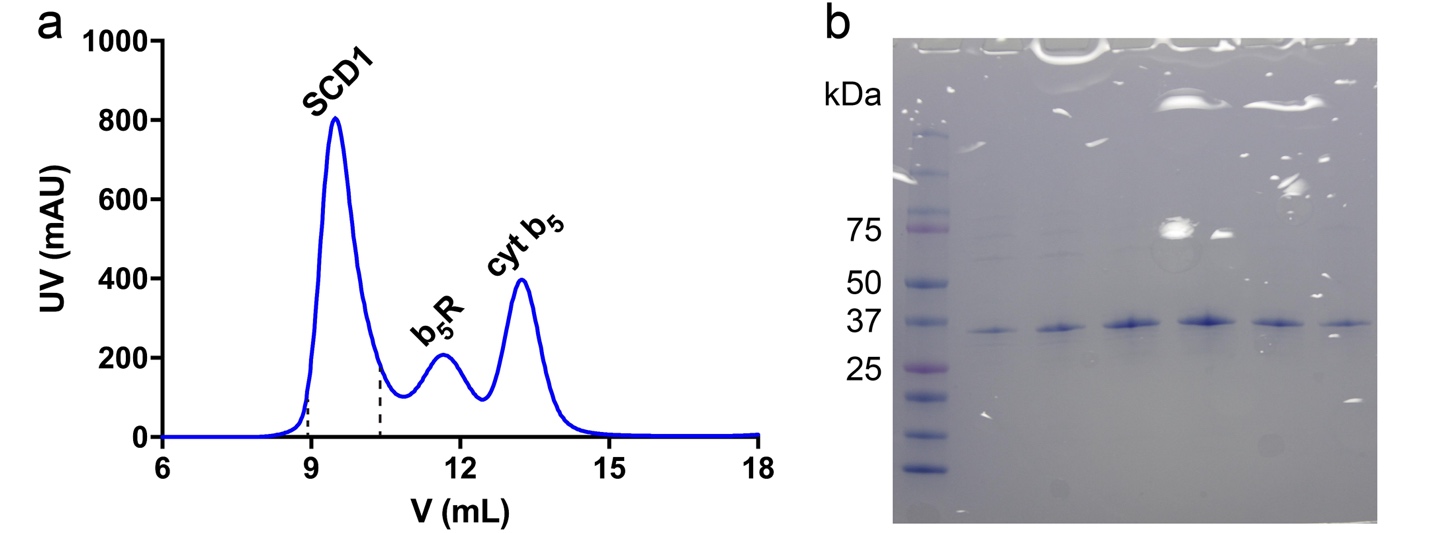
**

**Supplementary Figure 1.** Purification of SCD1 after reaction. (**a**) SEC profile of reaction mixture containing SCD1, soluble b_5_R and cyt b_5_ in Superdex 75 column. Vertical black dashed lines indicate the region of elution collected for SCD1. (**b**) SDS-PAGE gel image of the collected fractions in (**a**).

**
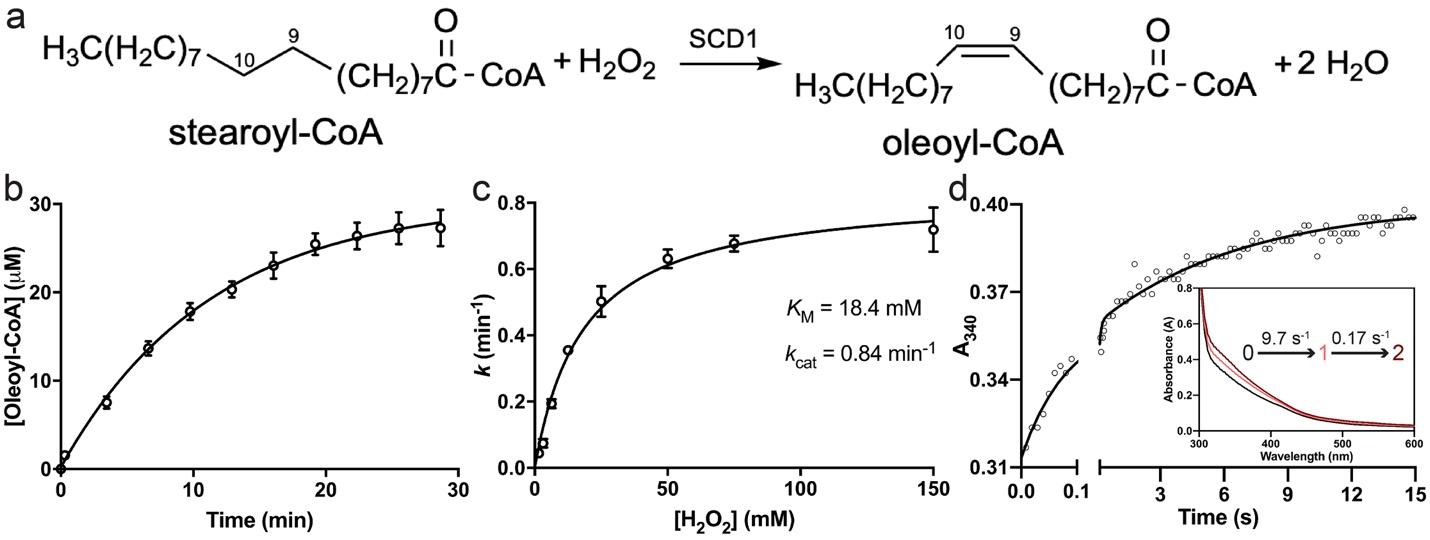
**

**Supplementary Figure 2.** Reactivity of SCD1 in the peroxide-shunt pathway with H_2_O_2_. (**a**) Overall reaction scheme of the peroxide-shunt activity in SCD1. (**b**) Time course of oleoyl-CoA production with H_2_O_2_. (**c**) Michaelis-Menten kinetics of the peroxide-shunt pathway. (**d**) Pre-steady-state kinetics of oxidation of the diiron center in SCD1 by H_2_O_2_. The inset shows the spectra of three species (black, pink, and red) deconvoluted from kinetics analyses.

**
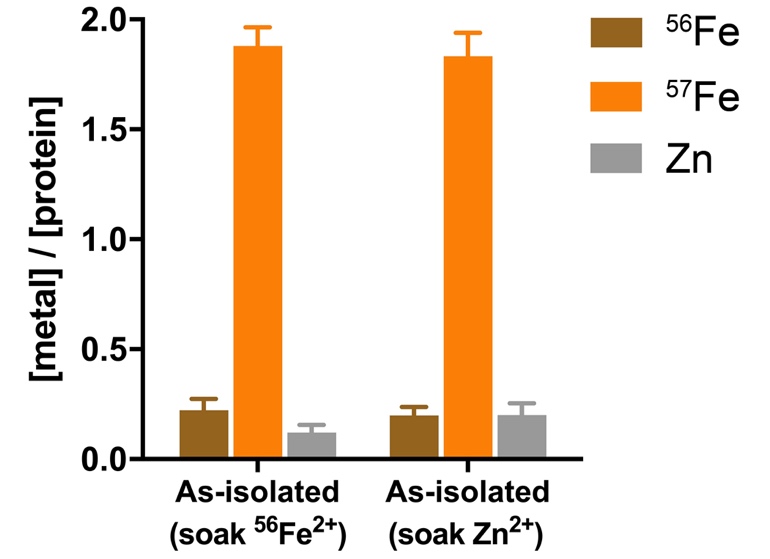
**

**Supplementary Figure 3.** Metal content analysis of ^57^Fe-enriched SCD1 soaked with ^56^Fe^2+^ or Zn^2+^ without initiating reaction.

**
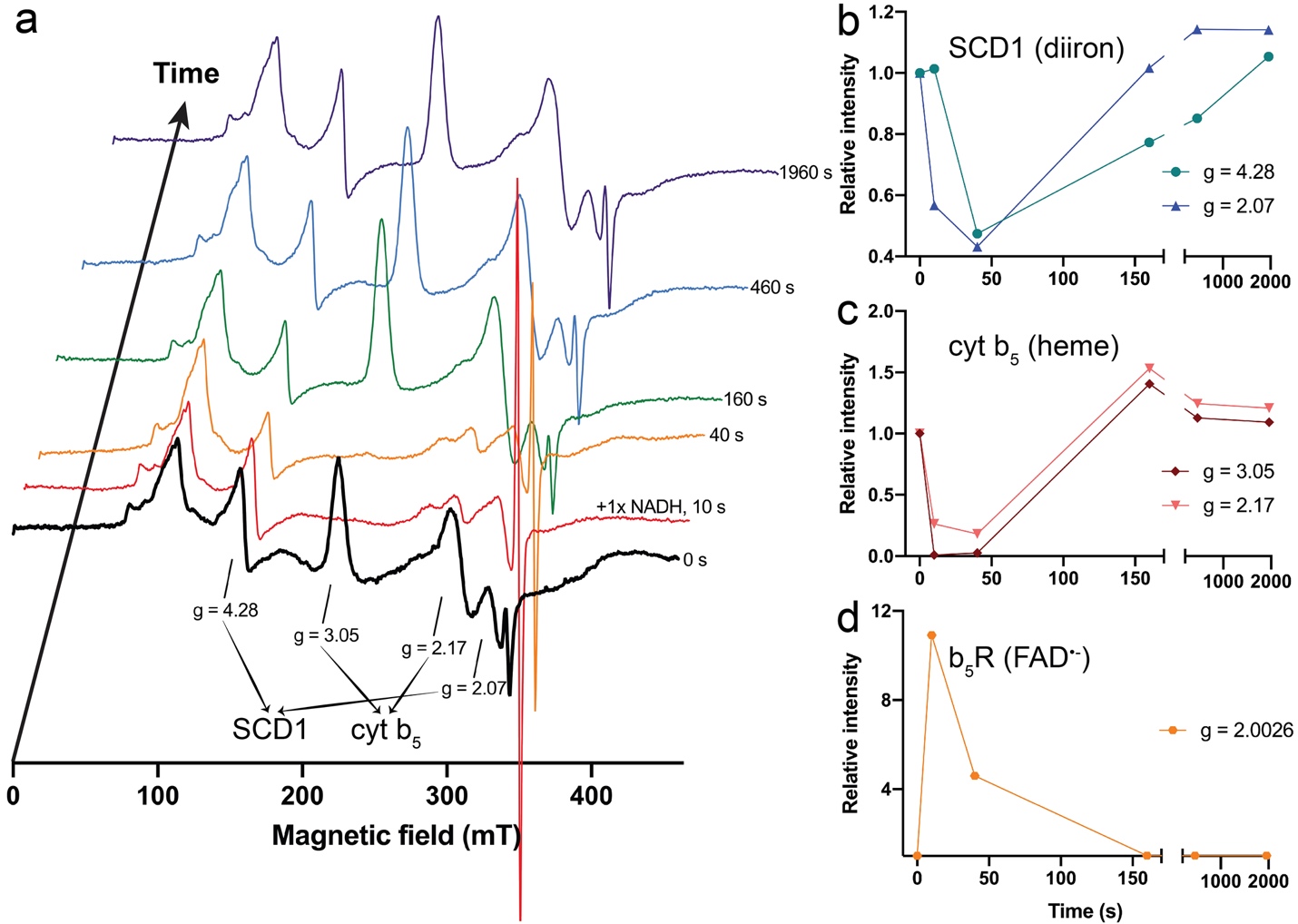
**

**Supplementary Figure 4.** Redox cycle of SCD1-cyt b_5_-b_5_R complex followed by EPR spectroscopy. (**a**) EPR spectra of the ternary complex at 10 K. Characteristic *g*-values of resting state SCD1 and cyt b_5_ are labeled. Time stamps to the right of each spectrum represent the incubation time after the addition of one molar equivalent (1×) of NADH. The large sharp signal at *g* ~2.0 popping up within 10 s is from the FAD**^-^·** in b_5_R. Time-dependent changes of relative intensities of the EPR species in: (**b**) SCD1 (*g* = 4.28 and 2.07); (**c**) cyt b_5_ (*g* = 3.05 and 2.17); and (**d**) FAD**^-^·** in b_5_R (*g* = 2.0026). The sharp signal at *g* ~2.0 from SCD1 at resting state is not considered due to its significantly smaller size compared to the strong signal from FAD**^-^·**.

**
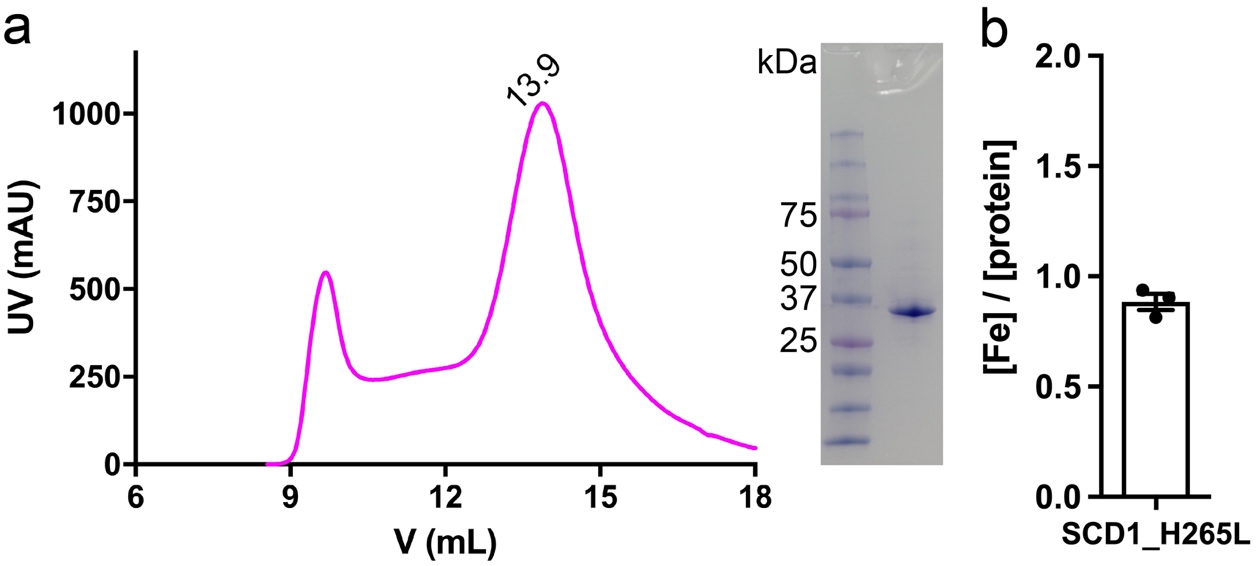
**

**Supplementary Figure 5.** Purification of SCD1_H265L. (**a**) Left: SEC profile of SCD1_H265L in Superdex 200 column. Right: SDS-PAGE gel image of peak fractions. (**b**) Fe content of SCD1_H265L.

**
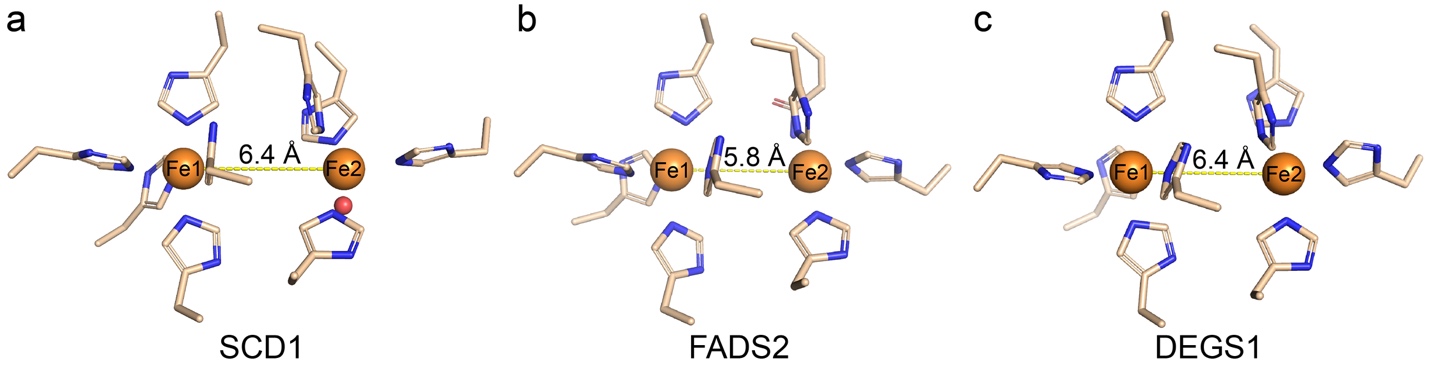
**

**Supplementary Figure 6.** Comparison of diiron centers in some mammalian membrane-bound enzymes. (**a**) Crystal structure of the diiron center in mouse SCD1 (PDB ID: 6WF2). AlphaFold2-predicted models of active sites in: (**b**) human fatty acid desaturase 2 (FADS2, UniProt ID: [O95864](https://www.uniprot.org/uniprotkb/O95864/entry)); (**c**) human sphingolipid Δ4-desaturase-1 (DES1, UniProt ID: [O15121](https://www.uniprot.org/uniprotkb/O15121/entry)). Fe ions are placed at coordination distances to ligand residues in the predicted models.
